# Clinical Significance of Hepsin and Underlying Signaling Pathways in Prostate Cancer

**DOI:** 10.3390/biom12020203

**Published:** 2022-01-25

**Authors:** Lucy Lu, Adam Cole, Dan Huang, Qiang Wang, Zhongming Guo, Wancai Yang, Jim Lu

**Affiliations:** 1 GoPath Laboratories, Buffalo Grove, IL 60089, USA; llu@gopathlabs.com (L.L.); dhuang@gopathlabs.com (D.H.); qwang@gopathlabs.com (Q.W.); zguo@gopathlabs.com (Z.G.); 2 TruCore Pathology, Little Rock, AR 72204, USA; adam@trucorepath.com; 3 Department of Pathology, University of Illinois at Chicago, Chicago, IL 60612, USA

**Keywords:** hepsin, prostate cancer, metastasis, survival, biomarker, target

## Abstract

The hepsin gene encodes a type II transmembrane serine protease. Previous studies have shown the overexpression of hepsin in prostate cancer, and the dysregulation of hepsin promotes cancer cell proliferation, migration, and metastasis in vitro and in vivo. The review incorporated with our work showed that hepsin expression levels were specifically increased in prostate cancer, and higher expression in metastatic tumors than in primary tumors was also observed. Moreover, increased expression was associated with poor outcomes for patients with prostate cancer. Using in silico protein–protein interaction prediction, mechanistic analysis showed that hepsin interacted with eight other oncogenic proteins, whose expression was significantly correlated with hepsin expression in prostate cancer. The oncogenic functions of hepsin are mainly linked to proteolytic activities that disrupt epithelial integrity and regulatorily interact with other genes to influence cell-proliferation, EMT/metastasis, inflammatory, and tyrosine-kinase-signaling pathways. Moreover, genomic amplifications of hepsin, not deletions or other alterations, were significantly associated with prostate cancer metastasis. Targeting hepsin using a specific inhibitor or antibodies significantly attenuates its oncogenic behaviors. Therefore, hepsin could be a novel biomarker and therapeutic target for prostate cancer.

## 1. Introduction

Prostate cancer is the most common cancer in men and the third-leading cause of cancer death in the United States. It was estimated that, in 2021, approximately 248,530 men would be diagnosed with prostate cancer and an estimated 34,130 would die from prostate cancer [1,2]. In the past several decades, investigative studies have made tremendous advances in the study of prostate cancer, in terms of uncovering the potential causes and underlying mechanisms of carcinogenesis, and providing targeted prevention and treatments, significantly improving quality of life and survival time [3,4,5,6]. However, numerous cases are therapy resistant, or become progressive, showing biochemical recurrence and metastasis, during active surveillance, resulting in worse prognoses [7]. Although the prostate specific antigen (PSA) has been widely used for early diagnosis and monitoring the recurrence of prostate cancer [8,9,10], PSA levels are not always consistent with the grades of cancers, the status of cancer progress, and cancer metastasis; additionally, PSA is not a therapeutic target. Therefore, novel biomarkers and therapeutic targets that can better serve clinics are in high demand. In recent years, a type II transmembrane serine protease called hepsin has attracted attention due to its overexpression and oncogenic functions in prostate cancer [11,12,13,14,15,16,17,18,19,20,21,22].

The hepsin (HPN, TMPRSS1) gene is located on human chromosome 19q13.11 and encodes a 45-KDa protein with 417 amino acids [23]. Although ubiquitous expression of hepsin has been reported in normal tissues, its mRNA is highly expressed in the liver and kidneys. Hepsin is physiologically involved in multiple cellular functions, including cell growth, blood coagulation, metabolism, the maintenance of cell morphology and membrane integrity, cleaving extracellular substrates, and contributing to the proteolytic processing of growth factors [13,24].

In hepatocytes, hepsin is physiologically overexpressed and activates hepatocyte growth factor (HGF) [25,26,27]; the latter binds to the hepatocyte growth factor receptor and regulates cell growth, cell motility, and morphogenesis. Interestingly, HGF’s expression levels are very low in other normal tissues and unchanged or downregulated in cancer tissues [28,29], and it can exert tumor-suppressive effects.

The increased expression of hepsin at both the mRNA and protein levels has been reported in some solid cancers, including prostate [11,13,14,18,30], ovary [31,32], kidney [33], and breast cancers [34,35,36]. Biological functional studies have demonstrated oncogenic roles for hepsin in the regulation of cancer cell proliferation, invasion, migration, and metastasis, in in vitro and in vivo model systems [24].

## 2. Hepsin’s Expression and Clinical Significance in Prostate Cancer

**Figure 1:**
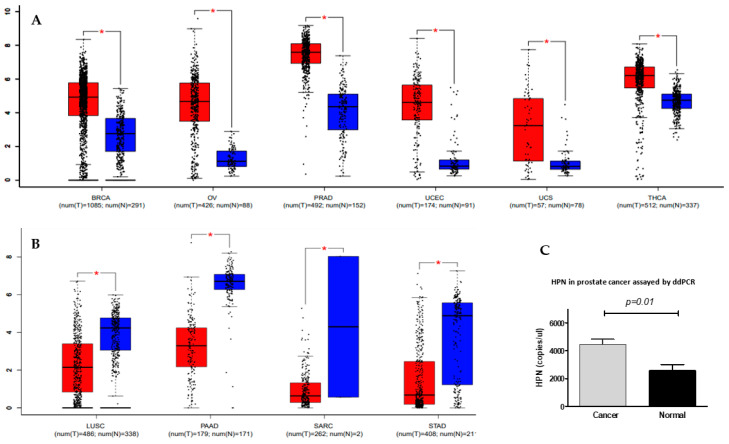
Differential expression of hepsin in solid cancers. (**A**) Hepsin expression levels were significantly increased in BRCA (breast invasive carcinoma), OV (ovarian serous cystadenocarcinoma), PRAD (prostate adenocarcinoma), UCEC (uterine corpus endometrial carcinoma), UCS (uterine carcinosarcoma), and THCA (thyroid carcinoma). Red represents cancer, and blue represents normal. * *p* < 0.05, tumor verse normal. (**B**) Hepsin expression levels were significantly decreased in LUSC (lung squamous cell carcinoma), PAAD (pancreatic adenocarcinoma), SARC (sarcoma), and STAD (stomach adenocarcinoma). Red represents cancer, and blue represents normal. * *p* < 0.05, tumor verse normal. (**C**) Reduced expression of hepsin was confirmed by digital droplet PCR (ddPCR) in prostate cancer.

Previous studies have shown that hepsin expression is increased in several types of solid cancers, such as prostate, breast, ovarian, and renal cancers, including different studies using small-sized human samples, as addressed above. By deeply mining the TCGA datasets with hundreds or even thousands of samples, we found that hepsin was differentially expressed in 33 types of cancer. Hepsin was upregulated in breast, ovarian, and prostate cancers, thymoma, uterine corpus endometrial carcinoma, and uterine carcinosarcoma, in comparison with associated normal tissues (Figure 1A). It is notable that hepsin expression in prostate cancer was the most significantly increased (492 cases of prostate cancer compared to 152 normal). By contrast, hepsin expression levels were downregulated in lung, pancreatic, and stomach cancers (Figure 1B). These findings were most likely consistent with previous studies on prostate cancer [11,13,14,18,30], ovarian cancer [31,32], kidney cancer [33], and breast cancers [34,35,36].

The significant increase in hepsin in prostate cancer was also observed in other studies [37,38,39,40,41,42]. Increased expression of hepsin mRNA was confirmed in our samples assayed by digital droplet PCR (Figure 1C). Analyzing the ovarian cancer and breast cancer TCGA datasets also confirmed the increases in hepsin mRNA levels in the ovarian and breast cancer tissues in comparison with their normal counterparts (Supplementary Figure S1A,B).

**Figure 2:**
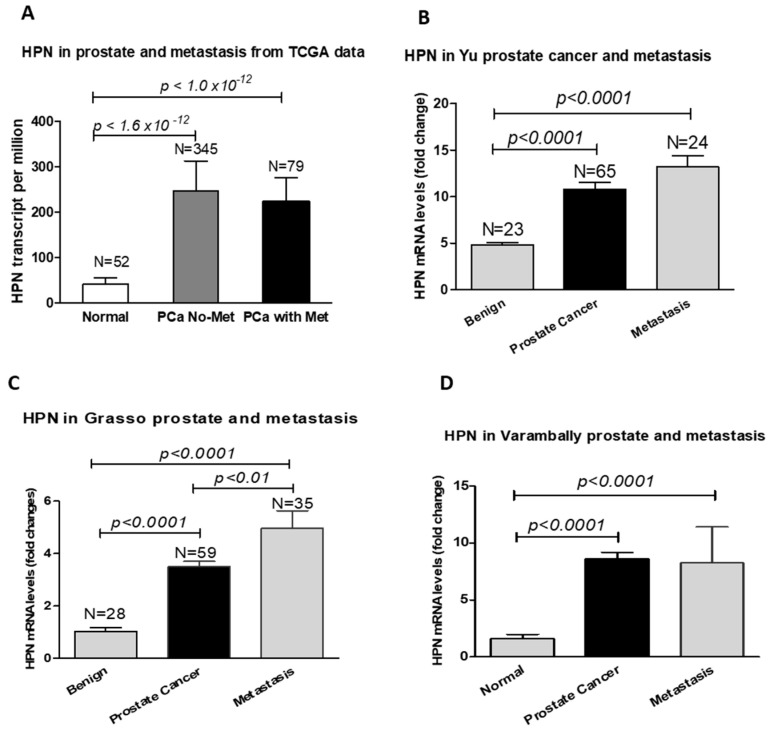
Hepsin mRNA levels were significantly increased in metastatic prostate tumors. (**A**) TCGA prostate cancer dataset. (**B**) The data from Yu’s study. (**C**) The data from Grasso’s study. (**D**) The data from Varambally’s prostate cancer study.

**Figure 3:**
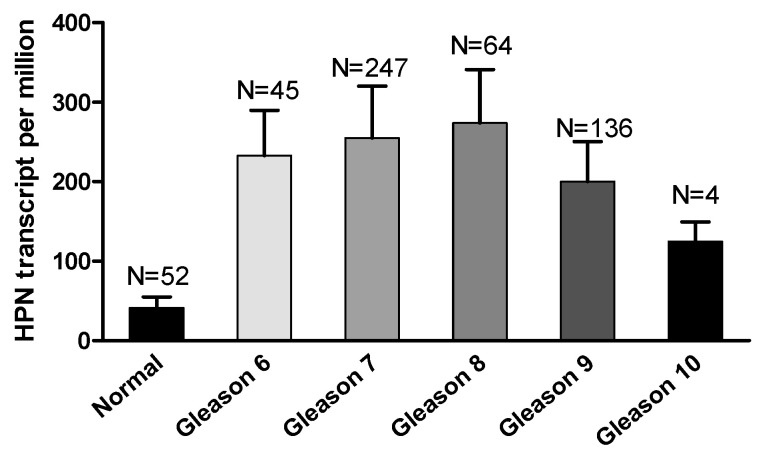
Hepsin expression levels were not associated with Gleason scores in prostate cancer.

Interestingly, hepsin mRNA levels were significantly increased in metastatic prostate tumors compared to the primary cancers [43,44,45], although the TCGA data for prostate cancer did not show a significant difference (Figure 2). Unexpectedly, the hepsin expression levels were not correlated with the prostate Gleason scores in the TCGA dataset (Figure 3), which was not consistent with previous reports showing that hepsin was upregulated by 34-fold in Gleason score grades 4 and 5 [18,45,46].

Additional analysis also showed that hepsin expression levels were much higher in renal [47] and colorectal metastatic cancers [48,49] in comparison with their primary cancers (Supplementary Figure S2A–C).

**Figure 4:**
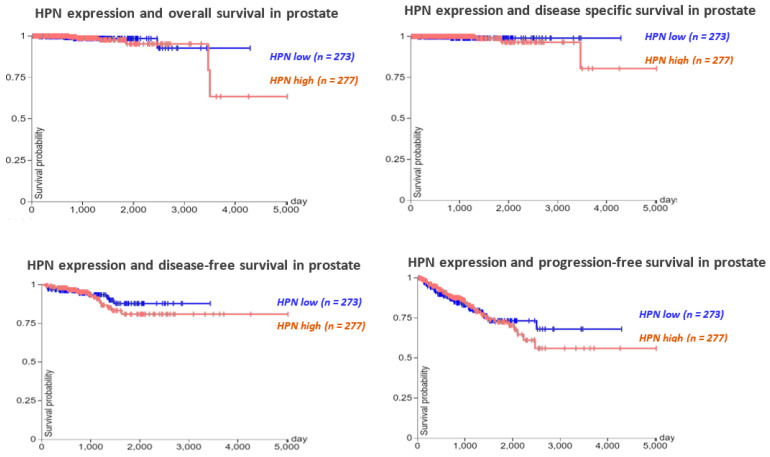
The association between HPN expression levels and overall survival, disease-specific survival, disease-free survival, and progression-free survival in prostate cancer (TCGA data, 550 cases of prostate cancer).

To determine the roles of hepsin in prostate cancer prognosis, the TCGA prostate cancer survival data (550 cases) were analyzed, showing that higher expression of hepsin was slightly associated with a shorter survival time, including overall survival, disease-specific survival, disease-free survival, and progression-free survival. (Figure 4). Similar results were observed for ovarian cancer, breast cancer, and gastric cancer upon analyzing 1435 cases of ovarian cancer (401 cases of low and 1034 cases of high expression), 1089 cases of breast cancer (279 cases of low and 810 cases of high expression), and 875 cases of gastric cancer (517 cases of low and 358 cases of high expression, respectively (Supplementary Figure S3A–C). By contrast, the higher expression of hepsin was positively associated with renal and hepatocellular carcinoma survival according to the analysis of 530 cases of renal cancer (131 cases of low and 399 cases of high expression) and 370 cases of liver hepatocellular carcinoma (136 cases of low and 234 cases of high expression) (Supplementary Figure S3D,E).

## 3. Oncogenic Functions of Hepsin and Underlying Molecular Regulation in Prostate Cancer

Both in vitro and in vivo studies have demonstrated oncogenic functions of hepsin in prostate cancer, such as promoting cell growth, cell proliferation, invasion, migration, and metastasis [12]. Although the underlying mechanisms are largely unknown, recent studies have reported the following functions and potential underlying mechanisms.

### 3.1. Hepsin Activates Macrophage-Stimulating Protein (MSP)

MSP is also known as macrophage stimulating 1 (MST1), an HGF-like protein [50]. This protein contains four Kringle domains and a serine protease domain; it stimulates ciliated epithelial cell motility in cooperation with the MST1 receptor tyrosine kinase, which is a member of the MET proto-oncogene family. Studies have demonstrated that plasma MSP levels are associated with prostate cancer progression, bone metastasis, and poor survival [51], and that MSP is required for prostate tumor growth in a TRAMP mouse model [52]. Ganesan et al. reported that hepsin could regulate the MSP/RON signaling pathway in tissue homeostasis and in disease pathologies (e.g., cancer and immune diseases) and, in fact, that the MSP/RON system could promote invasive tumor growth and suppress proinflammatory immune responses [53]. Thus, the oncogenic functions of hepsin could be mediated through activating MSP during prostate cancer progression and metastasis.

### 3.2. Hepsin Activates Pro-Urokinase-Type Plasminogen Activator (uPA)

Previous studies have revealed that pro-uPA is a substrate for hepsin and that the catalytic activities of hepsin are mediated through activating pro-uPA [54]. Indeed, hepsin could efficiently activate pro-uPA and then initiate plasmin-mediated proteolytic pathways at the tumor–stroma interface, leading to basement-membrane disruption and tumor progression [55]. Thus, hepsin’s enzymatic activity is linked to basement-membrane defects via the plasminogen/plasmin proteolytic pathway. In addition, hepsin, like matriptase, induces the potent destruction of the extracellular matrix through the cleavage of specific substrates. Hepsin also activates metalloproteinases and promotes matrix protein degradation [56]. The specific enzymatic activity of hepsin promotes cell motility and cancer cell metastasis.

### 3.3. Hepsin Cleaves Substrate Laminin-332

Laminin-332 is an extracellular matrix macromolecule, and the loss of laminin-332 expression is observed during human prostate cancer progression [57]. An association study showed that laminin-332 was a substrate of hepsin and was cleaved on the β3 chain by hepsin. An in vitro study showed that hepsin-cleaved laminin-332 enhanced the motility of DU145 prostate cancer cells. The direct cleavage of laminin-332 may be one mechanism by which hepsin promotes prostate cancer progression and metastasis, possibly by upregulating prostate cancer cell motility [57,58].

### 3.4. Hepsin Cleaves Stimulator of Interferon Genes (STING)

cGAS (cyclic GMP-AMP synthase) is a cytosolic DNA sensor that participates in an immune response against the invasion of microbial pathogens, and the activation of cGAS, in turn, stimulates the adapter protein STING, triggering interferon signaling [59,60]. Emerging evidence has demonstrated that the cGAS–STING pathway is an important mechanism of inflammation-driven tumor growth [61]. Thus, this pathway primarily functions as a tumor suppressor, and could also have tumor- and metastasis-promoting functions in the tumor microenvironment [59,61]. A very recent study showed that hepsin could cleave STING and suppress STING-mediated type I interferon induction and responses in hepsin-producing prostate cancer cell lines, dependent on the protease activity of hepsin [62], which may lead to increased susceptibility to cancer progression in addition to the vulnerability of hepatocytes to chronic viral infections.

### 3.5. Hepsin Cooperates with MYC

The first in vivo evidence showing an oncogenic role for hepsin was generated by characterizing a hepsin-transgenic mouse model, a model specifically overexpressing hepsin in the prostate, created by using a prostate-specific probasin promoter [12]. These mice exhibited normal cell proliferation and differentiation in the prostates, but the basement membrane was disorganized. Moreover, when the hepsin transgenic mice were cross-mated with the LPB-Tag 12T-7f models, the bi-transgenic mice showed significant tumor progression and metastases to the bone. These studies strongly suggested critical roles for hepsin in prostate cancer progression and metastasis, consistent with the results showing that higher levels of hepsin were found in patients with advanced prostate cancer. Further studies showed that hepsin/myc bigenic mice generated by crossing PB-hepsin mice with the PB-Hi-myc transgenic mouse model developed invasive adenocarcinoma at 4.5 months and higher-grade adenocarcinoma at 12–17 months of age. Moreover, the endogenous expression of hepsin was also upregulated as the tumors progressed in the PB-Hi-myc mice. These results indicate that hepsin and MYC cooperate during the progression to high-grade prostate adenocarcinoma [63].

### 3.6. Hepsin Interacts with miR-222–AKT Axis

The phosphatidylinositol 3-kinase (PI3K)/AKT pathway has been implicated in prostate carcinogenesis and metastasis [64]. The PI3K enzymes are primarily involved in the phosphorylation of membrane inositol lipids, thus mediating cellular signal transduction [65,66]. In prostate cancer, multiple factors cause AKT activation and translocation to the cytoplasm and nucleus, resulting in downstream target activation, functioning in survival, proliferation, progression, migration, and angiogenesis [67]. For example, PI3K/AKT signaling can cause the activation and upregulation of N-cadherin, and an increased level of N-cadherin is associated with angiogenesis and bone metastasis, suggesting that PI3K/AKT signaling may promote bone metastasis by regulating N-cadherin [68]. Moreover, PI3K/AKT signaling can target NF-kappa-B and lead to the activation of the bone morphogenetic protein signaling cascade, also promoting prostate cancer bone metastasis [69]. An in vitro study provided further evidence that hepsin interacts with the miR-222/AKT axis, promoting epithelial–mesenchymal transition (EMT) and cell invasion in prostate cancer [15].

### 3.7. Hepsin Is Activated by Oncogenic RAS

The Ras gene is well characterized. The Ras-encoded protein plays a causal role in human cancer by activating multiple pathways and promoting cancer growth and progression [70]. Oncogene-activating mutations, particularly amplifications, are found in about one-third of human cancers, leading to the activation of the RAFMEK–ERK signaling pathway [71]. Moreover, Ras destabilizes adherens junctions by downregulating E-cadherin and beta-catenin expression and disrupting tight-junction formation. Recently, Tervonen et al. reported that oncogenic Ras disrupted epithelial integrity by activating hepsin, disrupting epithelial cell integrity through the Ras-mediated deregulation of epithelial cell–cell and cell–matrix interactions and the cohesion of the epithelial structure. Therefore, hepsin is a critical protease for Ras-dependent tumorigenesis and early tumor dissemination [72].

### 3.8. Hepsin-Correlated Gene Expression and Correlation in Prostate Cancer

**Figure 5:**
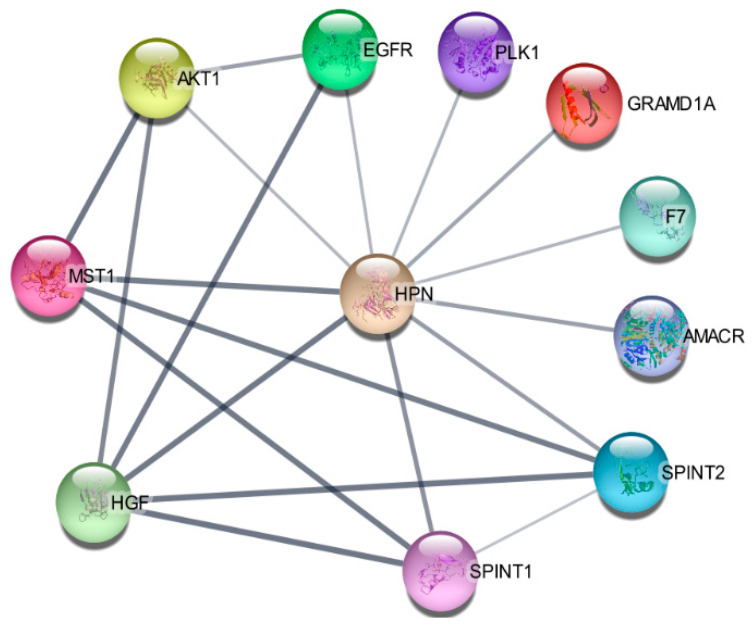
Hepsin protein–protein interaction (PPI) network.

**Figure 6:**
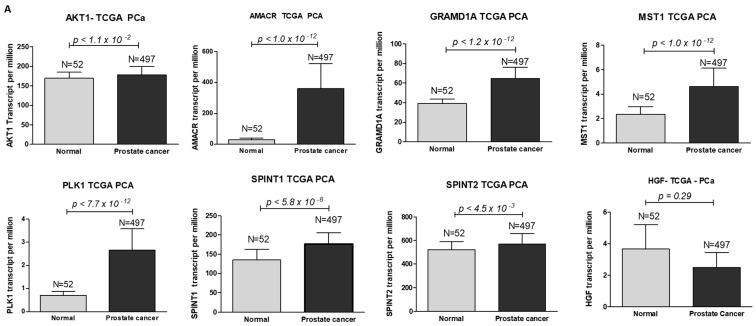

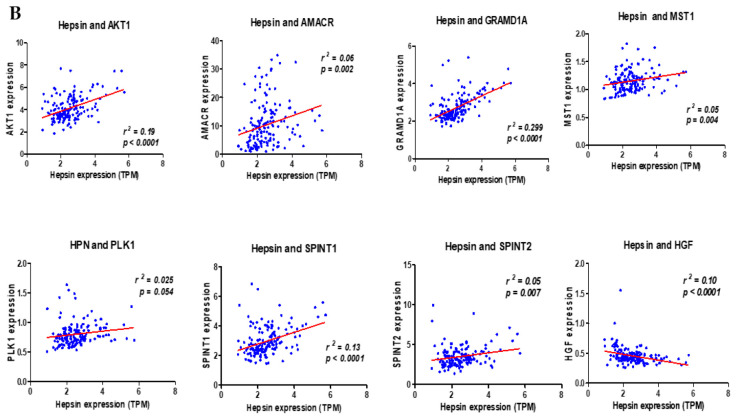
Hepsin PPI network genes’ expression levels and their correlation in prostate cancer. (**A**) Gene expression levels from TCGA data (497 cases of prostate cancer and 52 normal prostate tissues). (**B**) Gene correlation in prostate cancers from Taylor’s 155 cases of prostate cancer.

Using protein–protein interaction software, we noticed several known regulating genes and proteins in this network, including AMACR, AKT1, GRAMD1A, HGF, MST1, PLK1, SPINT1, and SPINT2 (Figure 5). Consistent with the hepsin expression levels in prostate cancer, these genes’ expression levels were also significantly increased in cancer tissues in comparison with normal prostate tissues (Figure 6A). Their expression levels were positively correlated with hepsin in prostate cancers according to an analysis of Taylor’s dataset [38] (Figure 6B). As described above, hepsin interacts with AKT1, HGF, MSP/MST1, etc., and the catalytic activities of hepsin are also regulated by serine protease Inhibitor Kunitz type 1 (SPINT1, HAI-1) and serine protease inhibitor Kunitz type 2 (SPINT2, HAI-2). Both proteins are potent specific inhibitors of the HGF activator and are thought to be involved in the regulation of HGF-mediated proteolytic activation. However, HGF expression is reduced in prostate cancer, compared to in normal tissues, and is negatively correlated with hepsin in prostate cancer (Figure 6B). Similar results were also observed in the TCGA prostate cancer dataset (Supplementary Figure S4).

## 4. Genetic Variants and Its Association with Prostate Cancer

Genetic variants are known to confer susceptibility to carcinogenesis and be associated with cancer progression and prognosis [73,74]. A Korean group reported that three single-nucleotide polymorphisms (SNPs) (rs45512696, rs2305745, and rs2305747) were significantly associated with the risk of prostate cancer based on a cohort study with 240 cases of prostate cancer and 223 control subjects [75]. Another case–control association study on hepsin variants and prostate cancer in a European ancestry cohort (590 cases and 576 controls) also showed significant allele frequency differences between cases and controls at five SNPs that are located contiguously within the hepsin gene [76]. Moreover, a major 11-locus haplotype is significantly associated with prostate cancer susceptibility, and one of the SNPs is associated with the Gleason score, showing the role of hepsin in tumor aggressiveness [76]. Our analysis, based on collective studies of a total of 7161 cases of prostate cancer, found that genomic alterations of hepsin (*p* < 0.0001; odds ratio, 3.95 (95% CI, 2.15–7.23)), particularly its amplification (*p* < 0.036; odds ratio, 4.16 (95% CI, 1.13–15.3)), were significantly associated with prostate cancer metastasis (1079 cases), compared to non-metastatic cases (6082 cases) (Table 1). These studies provide further evidence that hepsin is a potentially important candidate gene involved in prostate cancer formation and progression. By contrast, an American study based on 1401 prostate cancer and 1351 age-matched controls showed no association between hepsin SNPs or haplotypes and the risk of prostate cancer development, recurrence, or cancer death; thus, germline genetic variants of hepsin do not seem to contribute to the risk of prostate cancer and its prognosis [77].

## 5. Hepsin as a Biomarker in Liquid Biopsies

Since hepsin is specifically and significantly upregulated in prostate cancer tissues and metastatic cancer, it was anticipated to be a biomarker suitable for prostate cancer screening, diagnosis, or monitoring tumor progression using liquid biopsies. Beard et al. used enzyme-linked immunosorbent assays (ELISAs) to determine the circulating levels of hepsin in the serum for 424 patients with prostate cancer and found that the specificity was 89%, with a cut off >100 ng/mL determining hepsin positivity, in the active surveillance group. Among them, 18 patients showed biochemical recurrence, suggesting the diagnostic potential of circulating hepsin in prostate cancer [78]. Moreover, Roberts et al. reported that ejaculate-derived hepsin, PCA3, and miRNA, together with serum PSA, represented a better predictor of the status and risk of prostate cancer than PSA alone [79], showing the improved diagnostic and prognostic value of the use of a combination of different types of ejaculate biomarkers together with serum PSA.

## 6. Hepsin as a Therapeutic Target in Prostate Cancer

Several strategies for targeting hepsin have been developed [22], such as hepsin-specific inhibitors and antibodies, exhibiting preventive functions in reversing hepsin’s oncogenic functions in prostate cancer. For instance, a treatment with the hepsin inhibitor Kunitz domain-1 of prostate cancer DU145 cells could suppress cancer cell motility and further inhibit cancer cell progression and metastasis [80]. Chevillet et al. used a high-throughput system to identify and characterize a set of small-molecule inhibitors of hepsin, showing the attenuation of hepsin-dependent pericellular serine protease activity that was dose dependent, with limited or no cytotoxicity toward several types of cells in vitro [81]. Tang et al. reported that a small-molecule inhibitor, hepsin-13, targeting hepsin could inhibit bone, liver, and lung metastasis in a murine model of metastatic prostate cancer [82]. These findings suggest that the inhibition of hepsin with small-molecule compounds could provide an effective tool for the attenuation of prostate cancer progression and metastasis. In addition, a small-molecule inhibitor of uPA, WX-UK1, has also shown anti-tumor and anti-metastatic activity via downregulating hepsin expression [83]. Interestingly, Xuan et al. reported that neutralizing antibodies inhibited hepsin’s proteolytic activity in biochemical and cell-based assays and inhibited ovarian and prostate cancer invasion, but the neutralizing antibodies failed to inhibit the growth of prostate, ovarian, and hepatoma cell lines in culture [46]. Thus, hepsin may have a role in tumor progression, invasion, and metastasis but not in primary tumor growth, which was supported by a study in a hepsin transgenic mouse model, showing that the overexpression of hepsin in a mouse model of non-metastasizing prostate cancer had no impact on cell proliferation but caused disorganization of the basement membrane and promoted primary prostate cancer progression and metastasis to the liver, lungs, and bones [12].

## 7. Conclusions

Hepsin is specifically overexpressed in prostate cancer, and several studies have shown that the expression levels are even higher in metastatic tumors than primary tumors. In addition, the overexpression of hepsin in prostate cancer tissues is associated with a shorter survival time. The oncogenic functions of hepsin are mainly linked to proteolytic activities that disrupt epithelial integrity, and it regulatorily interacts with cell-proliferation, EMT/metastasis, inflammatory, and tyrosine-kinase-signaling pathways, promoting cancer cell motility and metastasis. Moreover, genomic amplifications of hepsin were significantly associated with prostate cancer metastasis. Targeting hepsin using a specific inhibitor or antibodies can attenuate its oncogenic behaviors. Therefore, hepsin could be a novel biomarker and a therapeutic target for prostate cancer.

The online datasets used for this study included: https://portal.gdc.cancer.gov, accessed on 2 September 2021; https://www.oncomine.org, accessed on 23 June 2021; http://gepia.cancer-pku.cn, accessed on 13 May 2021; https://www.cbioportal.org, accessed on 8 September 2021; https://www.proteinatlas.org, accessed on 6 July 2021; http://ualcan.path.uab.edu, accessed on 29 October 2021; https://kmplot.com/analysis, accessed on 21 October 2021.

## Figures and Tables

**Table 1:** Association between hepsin genomic alterations and metastasis in prostate cancer.

			Prostate Cancer without Metastasis			Prostate Cancer with Metastasis				
Total (%)-Mutation Types	Altered Numbers (%)	Non-Altered Numbers (%)	Sub-Total (%)-Mutation Types	Altered Numbers (%)	Non-Altered Numbers (%)	Sub-Total (%)-Mutation Types	Altered Numbers (%)	Non-Altered Numbers (%)	Odds Ratio (95% Confidence Interval)	*p* Value *
7161 (100)	44 (0.6)	7117 (99.4)	6082 (100)	26 (0.4)	6056 (99.6)	1079 (100)	18 (1.7)	1061 (98.3)	3.95 (2.15, 7.23)	<0.0001
Copy number alterations			Copy number alterations			Copy number alterations				
Amplification	23 (52.3%)		Amplification	10 (38.5%)		Amplification	13 (72.2%)		4.16 (1.13, 15.3)	0.036
Deletion	8 (18.2%)		Deletion	6 (23.1%)		Deletion	2 (11.1%)			NS
AAC	13 (29.5%)		AAC	10 (38.5%)		AAC	3 (16.7%)			NS
Sub-total	44 (100%)		Sub-total	26 (100%)			18 (100%)			

Abbreviation: AAC, amino acid change. * Comparison between without metastasis and with metastasis groups. NS, not significant.

## Supplementary Materials

The following supporting information can be downloaded at: https://www.mdpi.com/article/10.3390/biom12020203/s1, Figure S1: Hepsin expression levels in ovarian and breast cancers. TCGA data show that HPN mRNA levels were significantly increased in ovarian cancer (Figure S1A) and breast cancer (Figure S1B), Figure S2: Hepsin mRNA levels were significantly increased in metastatic renal cancer (Figure S2A) and colorectal cancers (Figure S2B,C), Figure S3: Higher expression levels of hepsin were correlated with better survival in ovarian cancer (Figure S3A), breast cancer (Figure S3B), and gastric cancer (Figure S3C) but were correlated with poor survival in renal cancer (Figure S3D) and hepatocellular carcinoma (Figure S3E), Figure S4: Protein–protein interactions (PPIs) and hepsin expression levels in the TCGA prostate cancer dataset showed a positive correlation among hepsin and AKT1 (Figure S4A), AMACR (Figure S4B), GRAMD1A (Figure S4C), MST1 (Figure S4D), SPINT1 (Figure S4E), SPINT2 (Figure S4F), and PLK1 (Figure S4G), but hepsin was negatively correlated with HGF (Figure S4H).
