# Supplementary material for: Clinical Significance of Hepsin and Underlying Signaling Pathways in Prostate Cancer
Source: Biomolecules. 2022 Jan 25;12(2):203. doi: 10.3390/biom12020203 (PMC8961580; doi:10.3390/biom12020203)

Supplementary figures legends

Supplementary figure S1. Hepsin expression levels in ovarian and breast cancers. TCGA data showed that HPN mRNA levels were significantly increased in ovarian cancer (Figure S1A) and breast cancer (Figure S1B).

Supplementary figure S2: Hepsin mRNA levels were significantly increased in metastatic renal cancer (Figure S2A) and colorectal cancers (Figure S2B, S2C).

Supplementary figure S3: The higher expression levels of Hepsin were correlated with better survival of ovarian cancer (Figure S3A), breast cancer (Figure S3B) and gastric cancer (Figure S3C), but were correlated with poor survival of renal cancer (Figure S3D) and hepatocellular carcinoma (Figure S3E), respectively.

Supplementary figure S4: Protein-protein interaction (PPI) and Hepsin expression levels in TCGA prostate cancer data set showed positive correlation among Hepsin and AKT1 (Figure S4A), AMACR (Figure S4B), GRAMD1A (Figure S4C), MST1 (Figure S4D), SPINT1 (Figure S4E), SPINT2 (Figure S4F) and PLK1 (Figure S4G), but was negatively correlated with HGF (Figure S4H).

Supplementary figure S1

Figure S1A

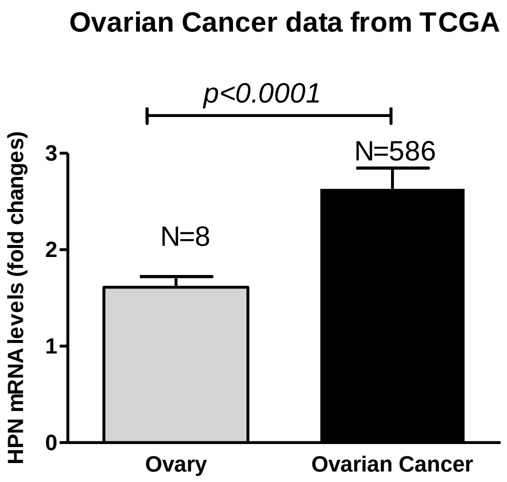

Figure S1B

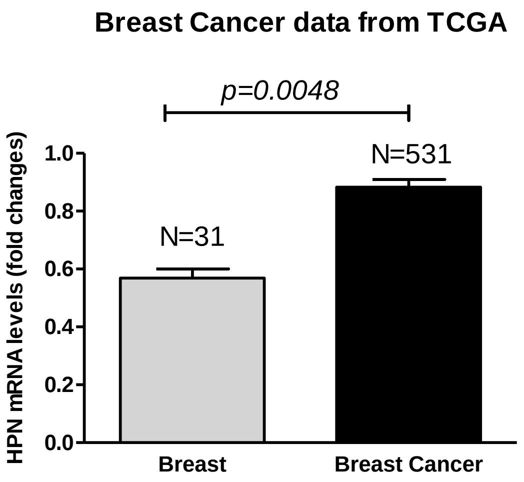

Supplementary figure S2

Figure S2A

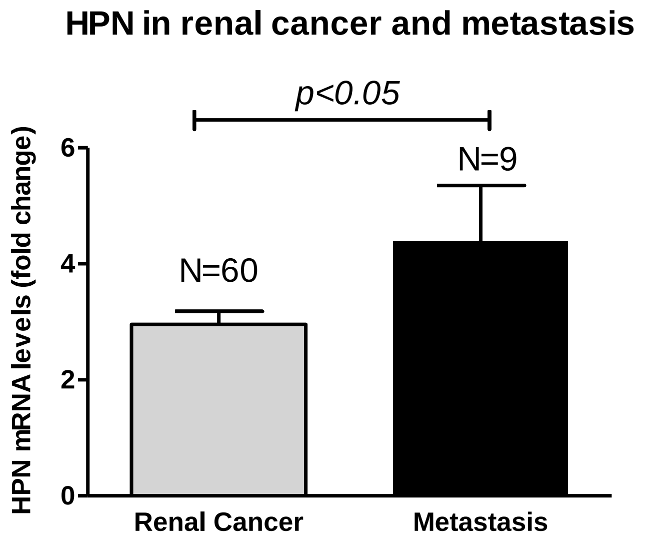

Figure S2B

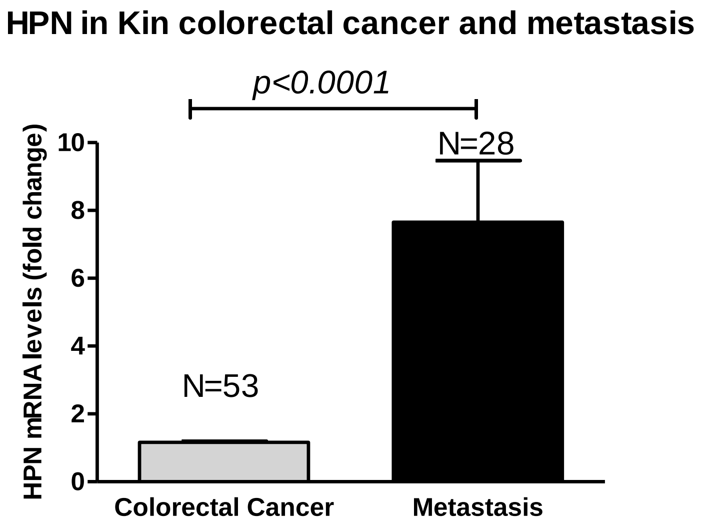

Figure S2C

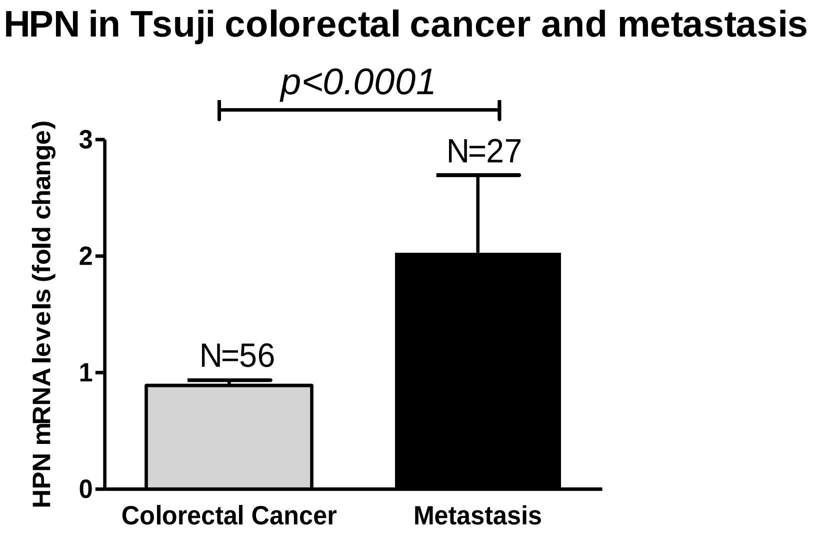

Supplementary figure S3A, S3B

Figure S3A

HPN expression levels and prognosis in ovarian cancer

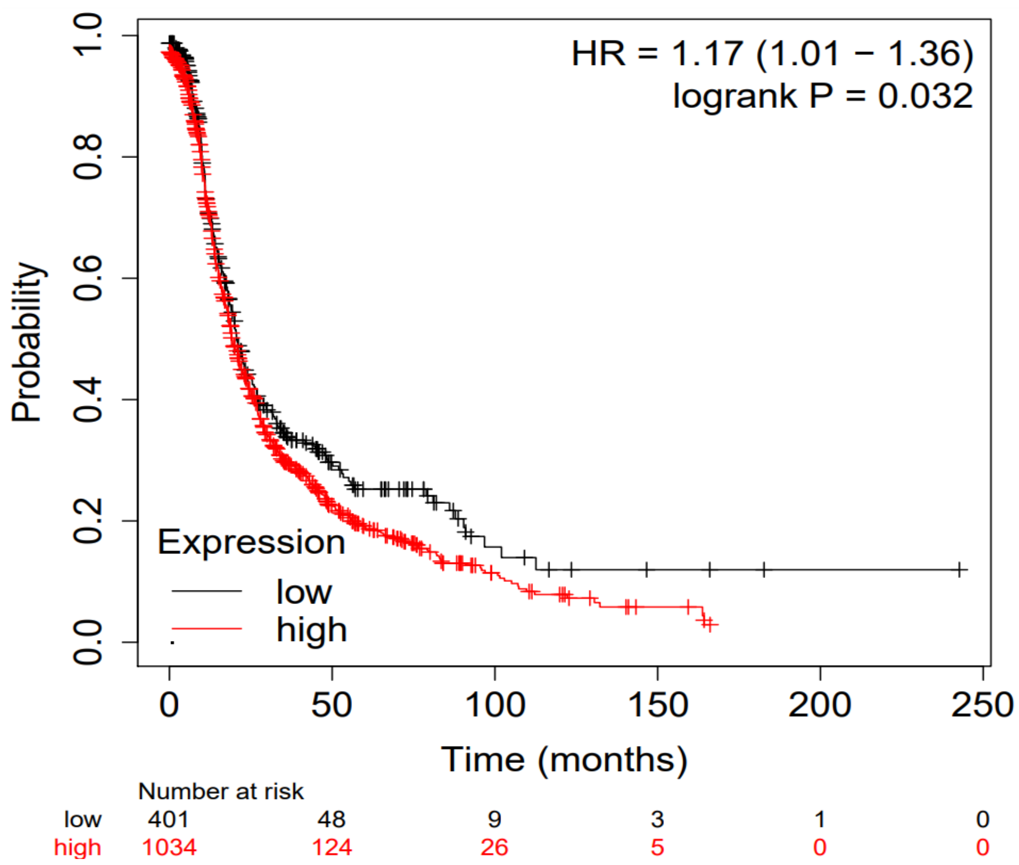

Figure S3B

HPN expression levels and prognosis in breast cancer

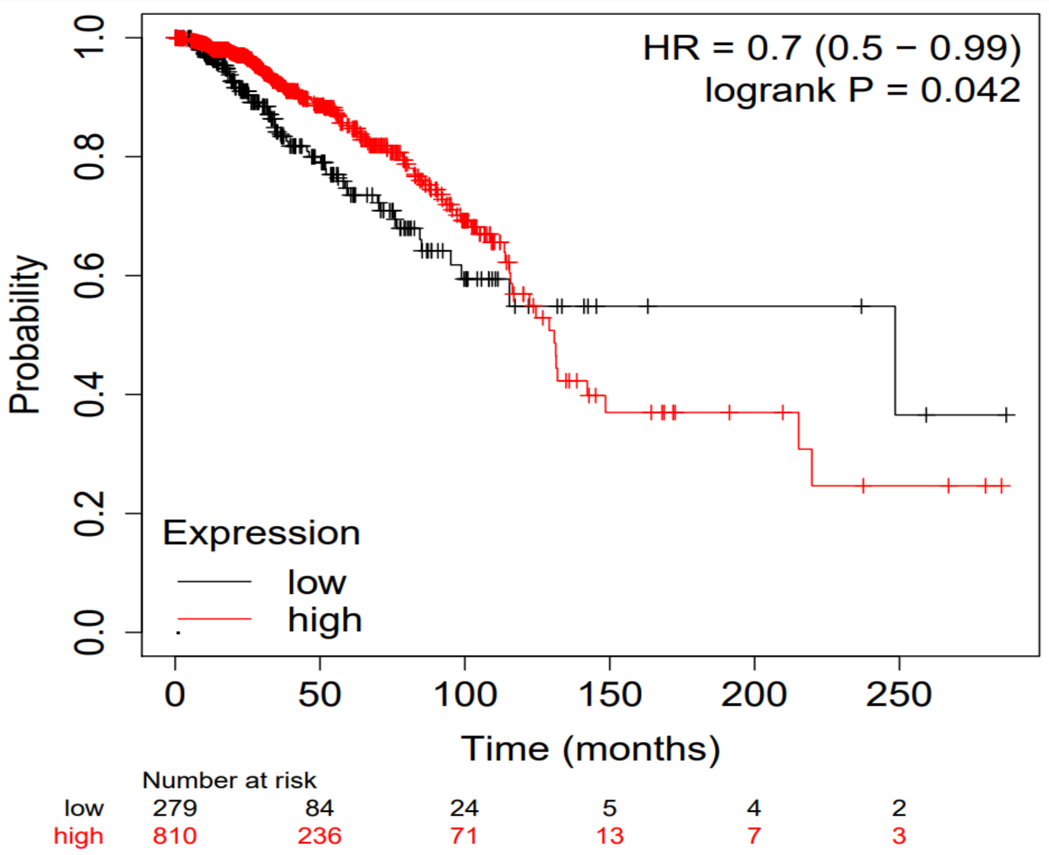

Supplementary figure S3C, S3D

Figure S3C

HPN expression levels and prognosis in gastric cancer

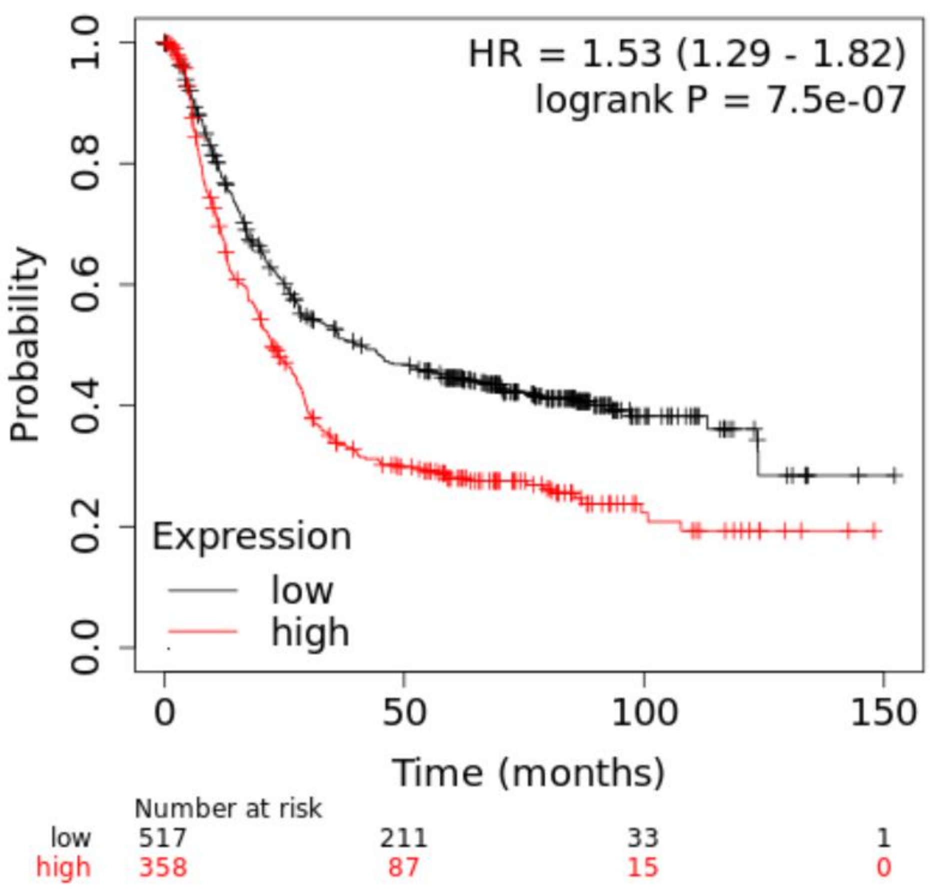

Figure S3D

HPN expression levels and prognosis in renal cancer

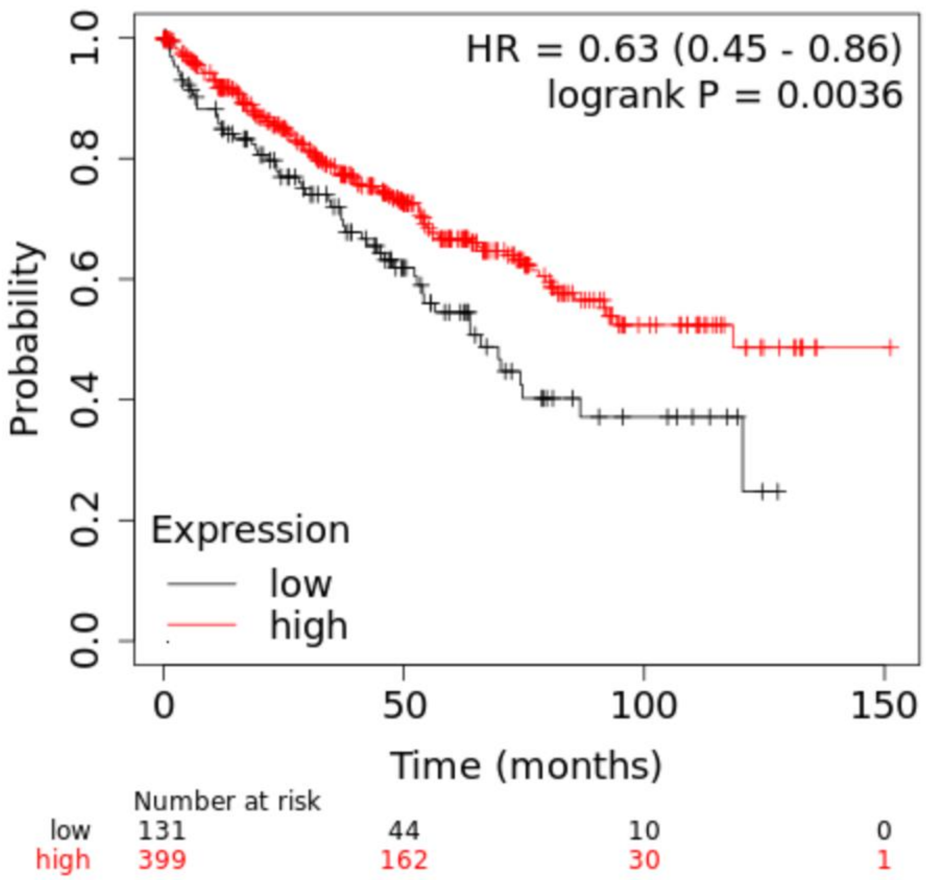

Supplementary figure S3E

Figure S3E

HPN expression levels and prognosis in hepatocellular carcinoma

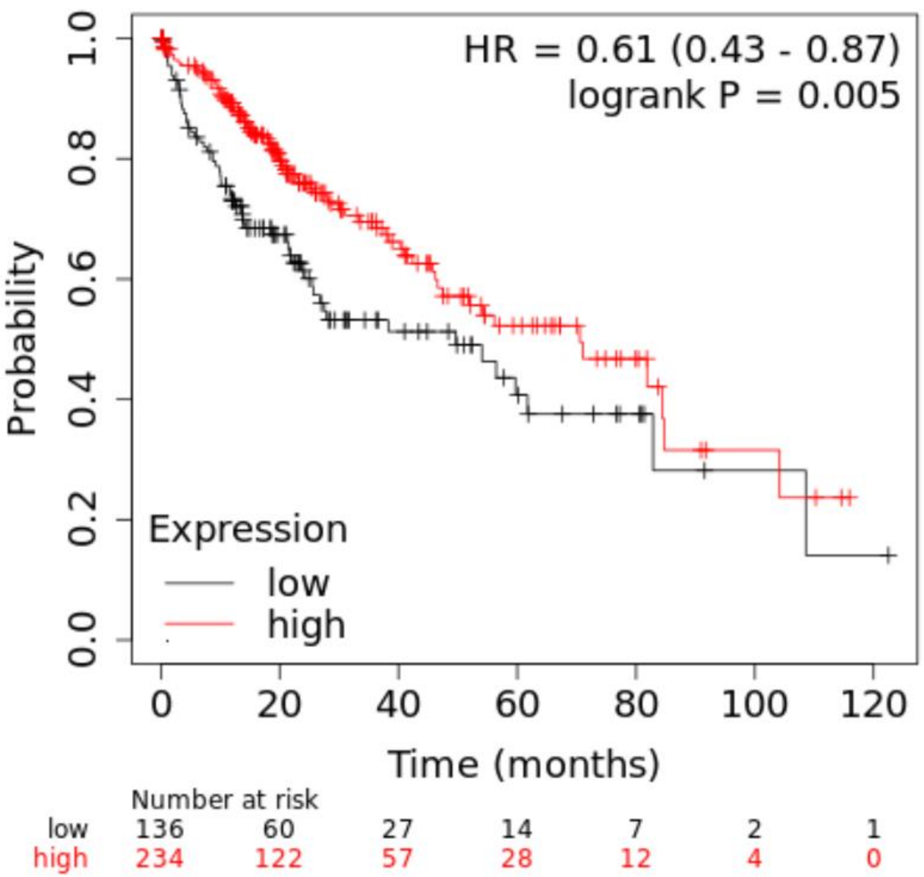

Supplementary figure S4A-S4D

Figure S4A

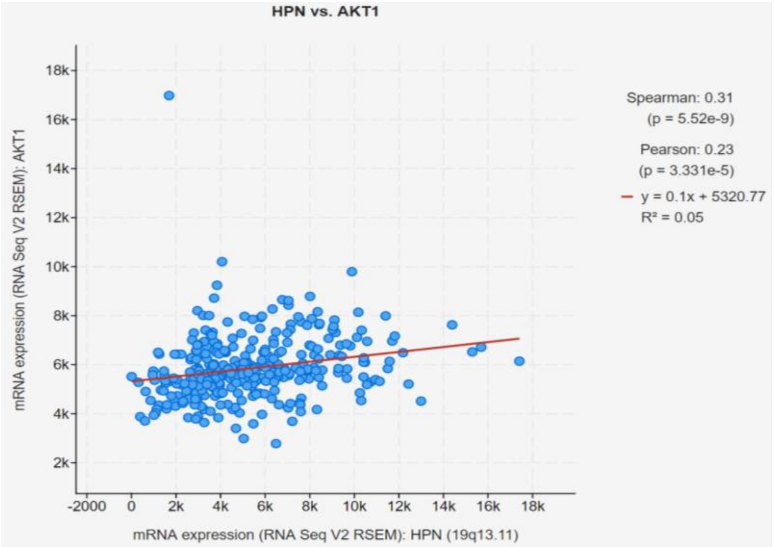

Figure S4B

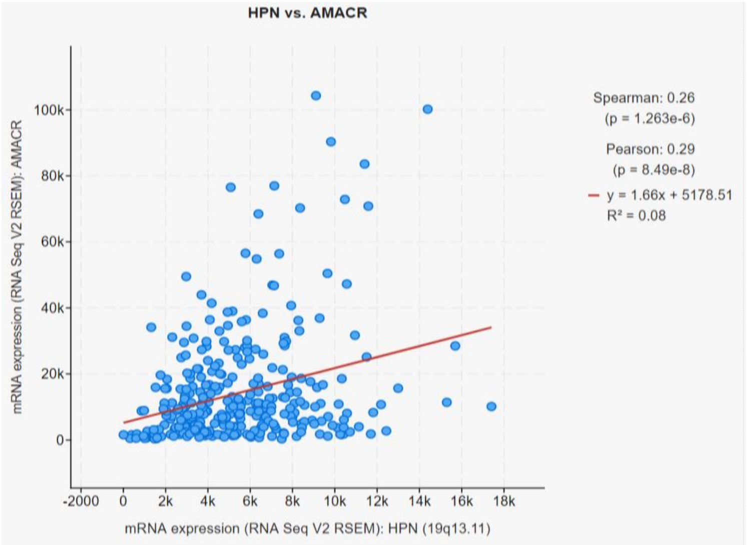

Figure S4C

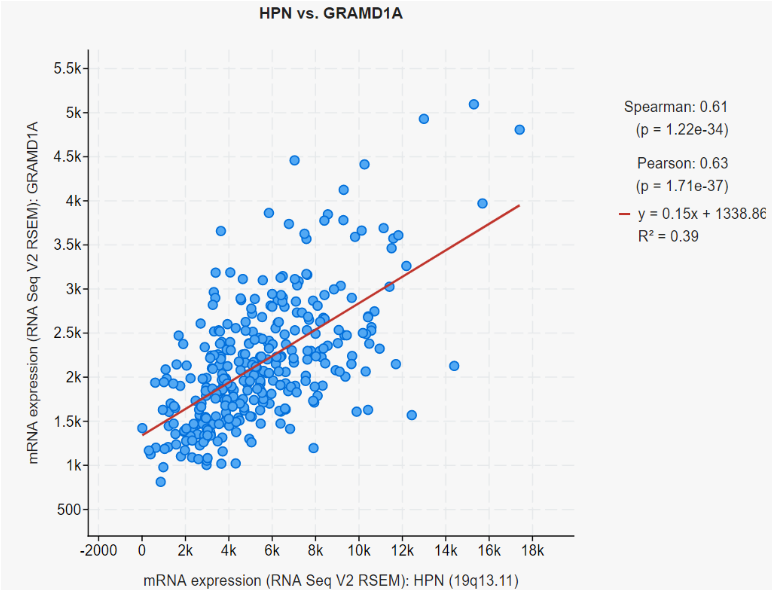

Figure S4D

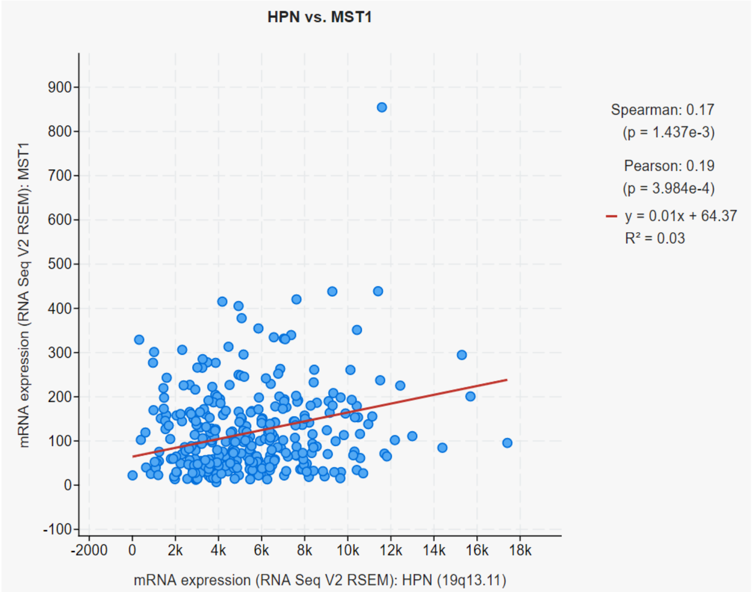

Supplementary figure S4E-S4H

Figure S4E

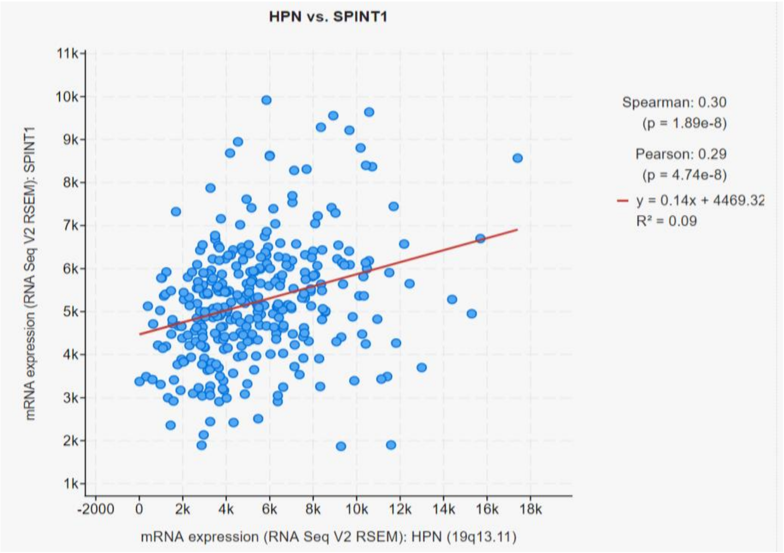

Figure S4F

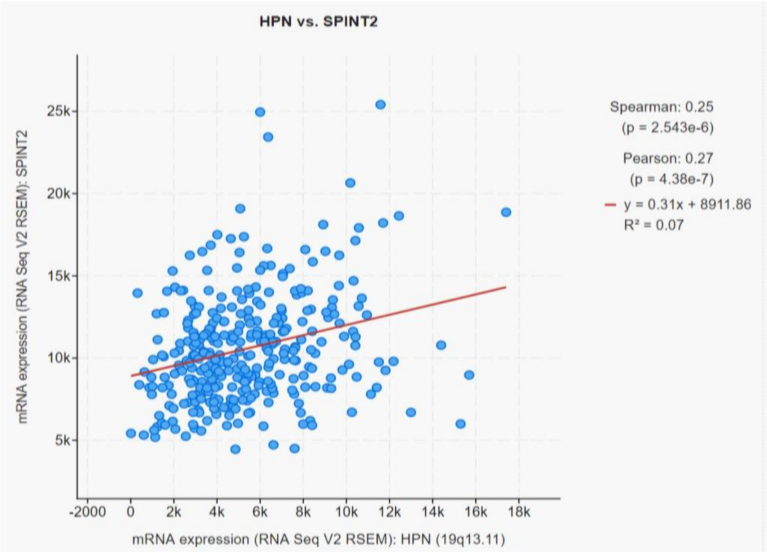

Figure S4G

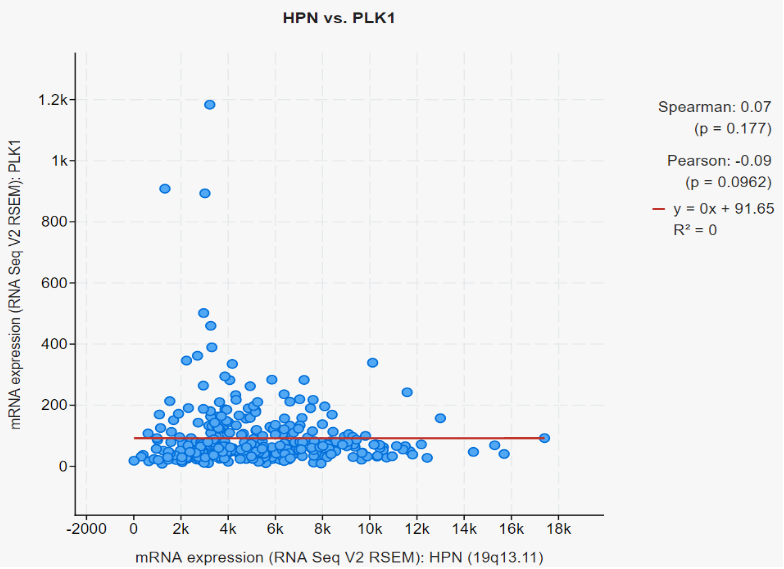

Figure S4H

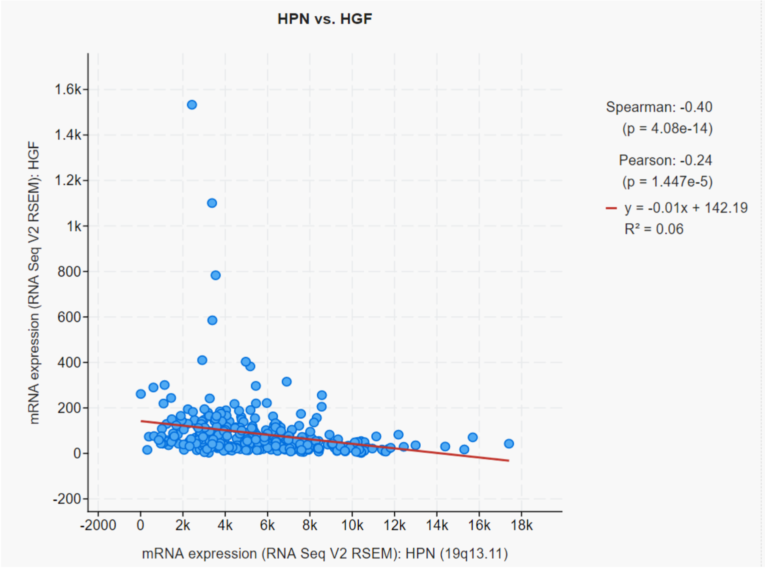

Supplement: Supplementary file 1 [file biomolecules-12-00203-s001.zip › biomolecules-1481426-supplementary.pdf]
